# Providing Instruction Based on Students’ Learning Style Preferences Does Not Improve Learning

**DOI:** 10.3389/fpsyg.2020.00164

**Published:** 2020-02-14

**Authors:** Beth A. Rogowsky, Barbara M. Calhoun, Paula Tallal

**Affiliations:** ^1^Department of Teaching and Learning, Bloomsburg University of Pennsylvania, Bloomsburg, PA, United States; ^2^Independent Researcher, Nashville, TN, United States; ^3^Center for Molecular and Behavioral Neuroscience, Rutgers University, Newark, NJ, United States; ^4^Center for Human Development, University of California, San Diego, San Diego, CA, United States

**Keywords:** learning styles, reading and listening comprehension, fifth grade, modality preference, auditory learners, visual learners, experimental design

## Abstract

Teachers commonly categorize students as visual or auditory learners. Despite a lack of empirical evidence, teaching to a student’s perceived learning style remains common practice in education (Pashler et al., 2009). Having conducted an extensive review of the literature, Pashler et al. (2009) noted, “...very few studies have even used an experimental methodology capable of testing the validity of learning styles applied to education” (p. 105). Rogowsky et al. (2015) published the first study following the experimental design prescribed by Pashler et al. Focusing specifically on the visual/auditory dichotomy, Rogowsky et al. (2015) examined the extent to which learning style predicts comprehension and retention based on mode of instruction. Their study has been noted as “The only study located through the systematic literature search across six different databases and the screening of more than 1000 records that was totally aligned with Pashler’s criteria” (Aslaksen and Loras, 2018, p. 3). The caveat to the 2015 study is that it was conducted with adult learners. The current study uses the same design and methodology as its predecessor, but on a school-aged population, making it the first of its kind. Consistent with earlier findings with adults, results failed to find a significant relationship between auditory or visual learning style preference and comprehension. Fifth graders with a visual learning style scored higher than those with an auditory learning style on listening and reading comprehension measures. As such, and counter to current educational beliefs and practices, teachers may actually be doing a disservice to students by using resources to determine their learning style and then tailoring the curriculum to match that learning style.

## Introduction

Learning styles-based education, specifically targeting auditory and visual learners, is common practice from kindergarten through post-secondary education (Lynch, 2015; Newton, 2015).

The underlying premise of learning styles is that teaching to a student’s preferred style results in optimal learning. For example, it is hypothesized that students classified as visual learners will recall more when content is presented in a visual format. Likewise, students classified as auditory learners will recall more when content is presented in an auditory format. Although it makes intuitive sense that students will learn best when taught in their preferred learning style, there have been multiple studies calling this methodology into question (Constantinidou and Baker, 2002; Kratzig and Arbuthnott, 2006; Massa and Mayer, 2006; Kassaian, 2007; Kolloffel, 2012; Hansen and Cottrell, 2013; Rogowsky et al., 2015; Knoll et al., 2017).

Despite the lack of evidence, adherence to learning styles hypotheses is globally pervasive. Dekker et al. (2012) surveyed 242 teachers from the United Kingdom (*n* = 137) and the Netherlands (*n* = 105) who were interested in applying neuroscientific findings in their classrooms. Given their high level of interest, it was predicted they would be current in effective research-based practices. Results showed that 93% of teachers from the United Kingdom and 96% of teachers from the Netherlands incorrectly agreed with the statement: “Individuals learn better when they receive information in their preferred learning style (e.g., auditory, visual, kinesthetic).” MacDonald et al. (2017) found similar results when they tested teachers, those with high exposure to neuroscience, and the general public with the same statement in the United States. Taken together, these results reveal the extent to which there continues to be a disconnect between empirical evidence and teaching practice. Misconceptions about learning styles continue to be widely held by teachers around the world.

Acknowledging the lack of empirical support, Pashler et al. (2009) published a comprehensive and influential review of the learning styles literature, specifically as it pertains to teaching. They called this interaction the meshing hypothesis which states that an individual learns better when taught in a mode of instruction (for example listening versus reading) that aligns with their preferred learning style (auditory, visual, respectively). Their review found no empirical evidence to support the meshing hypothesis. Since Pashler et al.’s (2009) review, a number of reviews have investigated learning styles and educator perceptions of their application. These have ranged from reviews of empirical studies on the effect of learning styles-based instruction on learning (Arbuthnott and Kratzig, 2015; Cuevas, 2015; Kirschner, 2017; Aslaksen and Loras, 2018), to studies on the persistence, prevalence, and disservice learning styles based instruction has had on education (Howard-Jones, 2015; Willingham et al., 2015; Kirschner, 2017). Like Pashler et al., these reviews found no support for learning styles, but no study was conducted with school-aged children.

In our review of the literature, we found two studies on learning styles conducted specifically with K-12 students (Martin, 2010; Mahdjoubi and Akplotsyi, 2012), but neither examined the effect of instructional mode on actual student achievement. Mahdjoubi and Akplotsyi had 151 elementary school students complete a child-friendly learning styles inventory: visual/aural/kinesthetic (VAK). All the children were then observed doing activities focused on different sensory styles (visual = photo safari, auditory = speech frequency, and kinesthetic = Global Positioning Systems). Mahdjoubi and Akplotsyi found that the children exhibited differences in their involvement during visual, auditory, and kinesthetic activities that were consistent with their assessed VAK learning style preference. However, Mahdjoubi and Akplotsyi did not assess student achievement or assess whether learning style differentially affected learning based on different modes of instruction. It is important to note that having learning preferences has never been called into question. What is under debate is whether learning in your preferred style yields greater academic achievement.

Martin (2010) also did not study the effect of learning styles-based instruction on achievement, but instead focused on the reliability of two learning style inventories. Martin administered two learning style inventories to 394 secondary students: Kolb’s Learning Style Inventory-2 (Kolb, 2005) and Honey and Mumford’s Learning Styles Questionnaire (Honey and Mumford, 1992). Despite the similar descriptors for the classifications between the two measures, no correlation between the two measures was found and there was a lack of construct validity for both inventories as well. Martin concluded, “Teachers would have as much information if they assigned the learning styles randomly to students rather than using the Kolb test” (p. 1586).

Given the lack of empirical studies, Pashler et al. described the experiment that would need to be conducted in order to conclude empirically that learning is significantly improved when individuals receive instruction matched to their learning style. First, individuals must be divided into groups on the basis of their learning style. Second, individuals from each group must be randomly assigned to receive one of multiple instructional methods. Finally, individuals must complete an assessment of the material that is the same for everyone. For the meshing hypothesis to be supported, the data analysis must reveal (1) that learning is optimal when individuals receive instruction in their preferred learning style and (2) the instructional method that proves most effective for individuals with one learning style is not the most effective method for individuals with a different learning style.

Using Pashler et al.’s prescribed experimental design and series of data analyses, Rogowsky et al. (2015) was unable to find support for the meshing hypothesis in adults. In their first experiment, adult participants’ auditory and visual learning styles were established based on a standardized adult learning style inventory (Building Excellence® online learning styles assessment inventory for ages 17 and older; Rundle and Dunn, 2010). Participants were then given an aptitude test in both listening and written formats (Gray Oral Reading Test-4th edition; Wiederholt and Blalock, 2000). Results showed no relationship between learning style preference (auditory, visual) and learning aptitude (listening comprehension, reading comprehension). In their second experiment, participants were randomly assigned to one of two instructional groups. The groups were presented with the same content, but in different instructional modes and then completed comprehension tests immediately following instruction and 2 weeks later. Results found no relationship between learning style, instructional method, and performance for either immediate comprehension or 2-week retention. Taken together, these experiments found no evidence to support learning styles-based instruction. However, it was noted that these results with adults may not generalize to children.

### The Current Study

Given that learning styles-based instruction is most highly targeted to the K-12 environment, coupled with the importance of verbal comprehension on educational outcomes, this study investigated the learning styles hypothesis, specifically as it pertains to the auditory/visual dichotomy in 5th graders. Implementing the methodology and analyses proposed by Pashler et al., we focused our study on the two most commonly targeted learning styles addressed in middle and secondary schools—visual and auditory.

Our investigation sought to answer the following research questions: (1) Is there a correlation between learning style and reading comprehension? The learning styles hypothesis predicts that visual learners would have higher reading comprehension scores. (2) Is there a correlation between learning style preference scores and listening comprehension? The hypothesis predicts that auditory learners would have higher listening comprehension scores. (3) Is there a correlation between the *difference* between reading and listening comprehension scores, and the *difference* between visual and auditory learning style scores? The hypothesis predicts that students with greater differences in their relative preference for visual or auditory learning would have greater differences between their reading and listening comprehension scores. (4) Do categorical learning style preferences predict comprehension? The hypothesis predicts that students will have higher scores in their learning style.

## Method

### Participants

Eligible participants included the entire population of 5th graders (ages 10–11 years) enrolled in a public middle school in rural Pennsylvania (*N* = 136). The actual participants were those students present for three consecutive days during which the study occurred (*n* = 125; 64 females/61 males). The school in which the study took place did not have a coordinated learning styles-based curriculum. Instructional methodology was decided upon by individual teachers so the students’ exposure to learning styles-based instruction varied. The study was approved and conducted in accordance with the standards of the university’s Institutional Review Board. Due to student absences, it was not possible to collect all data points from all students: learning styles scores were collected for 120 students; reading and listening comprehension scores were collected for 123 students; and 118 students had both learning styles scores and reading and listening comprehension scores. A power analysis showed that for an 80% chance of detecting a moderate effect size (Cohen’s *f*) of 0.15, with three predictors in the model and a type-I error rate of 0.05, the minimum sample size should be 76 students. Since the relevancy of this study concerns the use of learning styles at the classroom level, anything less than a moderate to large effect size would not be educationally meaningful or reliable.

### Learning Styles Assessment

Rundle and Dunn learning styles inventory, LSCY (Learning Style: The Clue to You!), was used because it is specifically designed for students aged 10–13 years. In addition, it has been referred to as the most popular by Pashler et al. (2009) and recommended for use in teacher education textbooks (Lynch, 2015). It is self-administered online requiring 20–25 min for completion and assesses individual learning styles based upon six domains: perceptual, psychological, environmental, physiological, emotional, and sociological. We focused on the visual and auditory learning styles which were included in the perceptual domain. The perceptual domain is subdivided into four elements: auditory, visual, tactile, and kinesthetic. We focused on the auditory and visual learning styles. Test–retest reliability coefficients for visual equaled 0.73; the coefficient for auditory equaled 0.92 (Dunn and Burke, 2005). For each learning style, individuals are rated from 1 (weak) to 5 (strong). We did not collect data related to other types of learning styles such as kinesthetics, conformity, motivation, and persistence.

The LSCY provides personalized reports to convert an individual’s numerical score into instructional prescriptions. For example, participants who score *preference* or *strong preference* (4 or 5) on the “Learns by Seeing” as well as *it depends, preference,* or *strong preference* (3, 2, 1) on the “Does Not learn by Listening” are instructed to use the visual modality and are classified as visual learners. In contrast, participants who score *preference or strong preference* (either a 4 or 5) on the “Learns by Listening” and *it depends, preference,* or *strong preference* (3, 2, 1) on the “Does Not learn by Seeing” are instructed to use the auditory modality and are classified as auditory learners. If a student is classified as an auditory learner, the student receives the following recommendations: “You often learn the things you really concentrate on by listening. Because of that, you remember much of what you hear during a lecture or when your teacher teaches a lesson by talking. … If your teacher requires that you read material first, explain that you need to hear new and difficult information before you read about it.”

### Instruction

Students’ comprehension was measured using a modified version of the fourth edition of the Gates MacGinitie Reading Test (GMRT; MacGinitie et al., 2010). The GMRT is a standardized, norm-referenced reading test with an internal consistency reliability for 4th and 5th grade ranging from 0.92 to 0.93. There are two forms for each grade (S and T) allowing multiple administrations. Alternate-form reliability at those grades is 0.86. After pilot testing passages from Grade Level 4 and 5 tests with fifth graders, Passages 1–11 from both forms (S and T) of Grade Level 4 and Passages 9 and 11 from both forms (S and T) of Grade Level 5 were selected for use in this study. Each passage contained 65–147 words and was followed by three to six comprehension questions.

Using those 13 passages, four measures were created: two contained the 13 selected passages from form S of the GMRT, and two contained the 13 selected passages from form T. One version of each form was written to be read, and the other form was recorded by a professional audiobook narrator to be listened to. As per the protocol designed by Pashler et al. (2009) to assess the learning styles hypothesis, individuals must complete an assessment that is the *same* for all participants. Therefore, following each passage, the GMRT questions were presented in the written form.

To assess listening comprehension, each participant used earbuds to listen to one of the forms of the GMRT. Immediately after listening to each passage, each participant answered the comprehension questions related to that passage. The listening assessment will be referred to as the Listening Aptitude Test (L-AT). To assess reading comprehension, immediately after reading each passage, participants answered the comprehension questions related to that passage. The reading assessment will be referred to as the Reading Aptitude Test (R-AT).

The assessments were presented on the students’ iPads. Each of the participants in this study was tested on *both* the L-AT and the R-AT using different GMRT forms (S or T). The form used for the L-AT and the R-AT and the order of presentation were both counterbalanced. Participants could not go back to a passage after completing it and could not proceed prior to answering a question.

### Procedure

The study occurred in the students’ language arts classroom at the beginning of the school year. All students received the LSCY as well as instruction in the listening and the reading conditions. For counterbalancing purposes, students were randomly assigned to one of four groups: two instructional modalities (auditory, visual) and two test forms (S and T). Each group received a different test form/modality combination on the first day and, in a crossover design, the opposite test form/modality on the second day. This counterbalanced the order of instruction across participants to mitigate order effects. On Day 3, students completed the learning style inventory. The participants were not informed about the purpose of the study, nor were they given feedback on their assessments or the results of the learning styles inventory.

## Results

The goal of this study was to determine whether there was an interaction between learning style preference (auditory, visual) and the modality of the most effective instruction. Support for the learning styles hypothesis would show that the instructional method that is best for individuals with one learning style is not the most effective method for individuals with a different learning style.

### Equivalence of the Comprehension Forms

**Figure 1:**
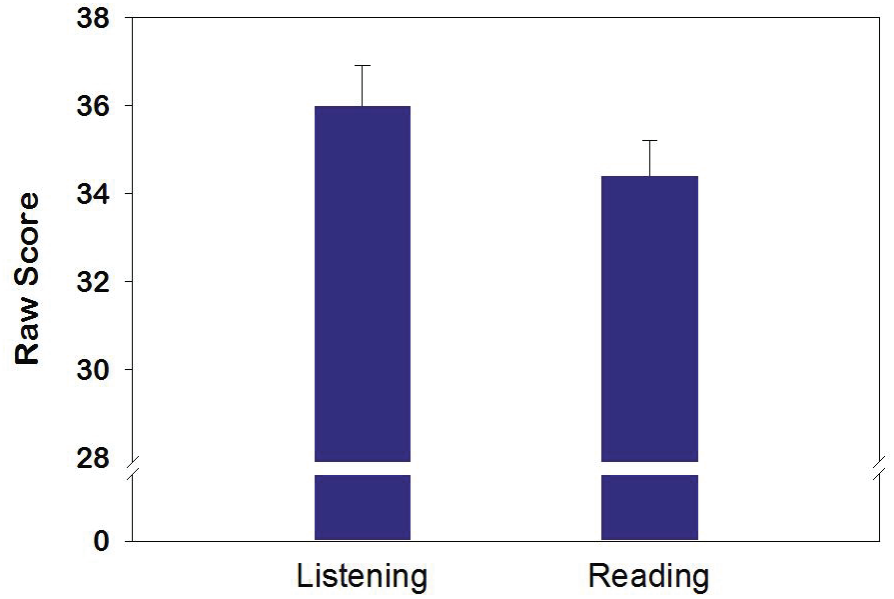
The students’ listening assessment scores were significantly greater than their scores on the reading assessment (*p* = 0.03).

Comprehension scores were checked for meeting the assumptions of an ANOVA. Focusing on scores that were above chance (*n* = 112) resulted in scores meeting all assumptions. A 2 (Form S, Form T) × 2 (instruction = reading, listening) between-subjects factorial ANOVA was calculated to evaluate the equivalence of the questions on the two forms (*N* = 112). A significant main effect for instruction was found (*F*(1, 110) = 4.92; *p* = 0.03) with students performing better when listening (*M* = 36.0, *SE* = 0.9) than reading (*M* = 34.4, *SE* = 0.8; see Figure 1). This corresponds to an effect size of *η*^2^ = 0.04, which is considered small. There was no effect of form (*F*(1, 110) = 3.83; *p* = 0.053), indicating that the two forms were not different. Additionally, there was no form by instruction interaction (*F*(1, 110) = 1.87; *p* = 0.17).

### Correlation Analyses

The relationship between learning style and comprehension was evaluated by a series of correlation analyses on the 107 students with both comprehension and LSCY scores. LSCY scores were based on the 5-point LSCY scoring system. The comprehension scores met the assumptions for a correlation analysis. To evaluate whether students with stronger visual preferences had better reading comprehension, a Pearson correlation coefficient was calculated. The weak correlation between the R-AT and LSCY visual score was not significant (*r* = 0.07, *n* = 107, *p* = 0.48, two-tailed). To evaluate whether students with stronger auditory preferences had better listening comprehension, a Pearson correlation was calculated. A significant, but weak, *negative* correlation was found between the L-AT and LSCY auditory score (*r* = −0.22, *n* = 107, *p* = 0.02, two-tailed). In accordance with Pashler’s model, it is important to consider the relative performance on the two comprehension tests. To assess this, a Pearson correlation coefficient was calculated for the relationship between the difference between reading comprehension and auditory comprehension (whereby positive scores indicate better reading comprehension), and the difference between the visual learning style preference and auditory learning style preference (whereby positive scores indicate visual learning style preference). The weak correlation was not significant (*r* = 0.05, *n* = 107, *p* = 0.62, two-tailed) indicating that the relative comprehension score was not related to the relative learning preference score.

### Analyses Using Categorical Learning Style Variables to Predict Comprehension: Implementing the Pashler Method

Pashler et al.’s (2009) suggested methodology for testing the meshing hypothesis requires participants to be categorically classified into two discrete learning styles (auditory learners or visual learners). Following this methodology, students were divided into groups based on their learning styles. Participants were limited to those who had a strong auditory and indifferent or weak visual learning style (*n* = 12; 11%) and those who had a strong visual and indifferent or weak auditory learning style (*n* = 22; 21%). Seventy-three students (68%) had similar preferences for auditory and visual learning styles: 47 had identical scores in their auditory and visual learning style (strong in both styles, *n* = 24; indifferent/weak in both styles, *n* = 23), the auditory or visual learning style for the other 26 students differed only by 1 or 2 points (strong in both styles *n* = 22; indifferent/weak in both styles *n* = 4).

A mixed-design two-way ANOVA was calculated examining the effects of learning style groups (auditory, visual) on the L-AT and R-AT scores to determine whether learning style (auditory, visual) predicts listening or reading comprehension (*n* = 34). Students’ L-AT and R-AT scores met the assumptions for an ANOVA including normality and homogeneity of variances. There was a significant main effect of learning styles (auditory vs. visual; *F*(1, 32) = 12.92; *p* = 0.001) with an effect size of *η*^2^ = 0.29, indicating that students in one learning style group (visual: *M* = 39.07, *SE* = 1.73) performed significantly better than those in the other learning style group (auditory, *M* = 28.58, *SE* = 2.35). There was not a significant main effect of instruction (*F*(1, 32) = 1.15, *p* = 0.29), nor an instruction by learning styles interaction (*F*(1, 38) = 1.16; *p* = 0.29). These results indicate that visual learners were significantly better at *both* listening and reading comprehension, as compared to auditory learners.

**Figure 2:**
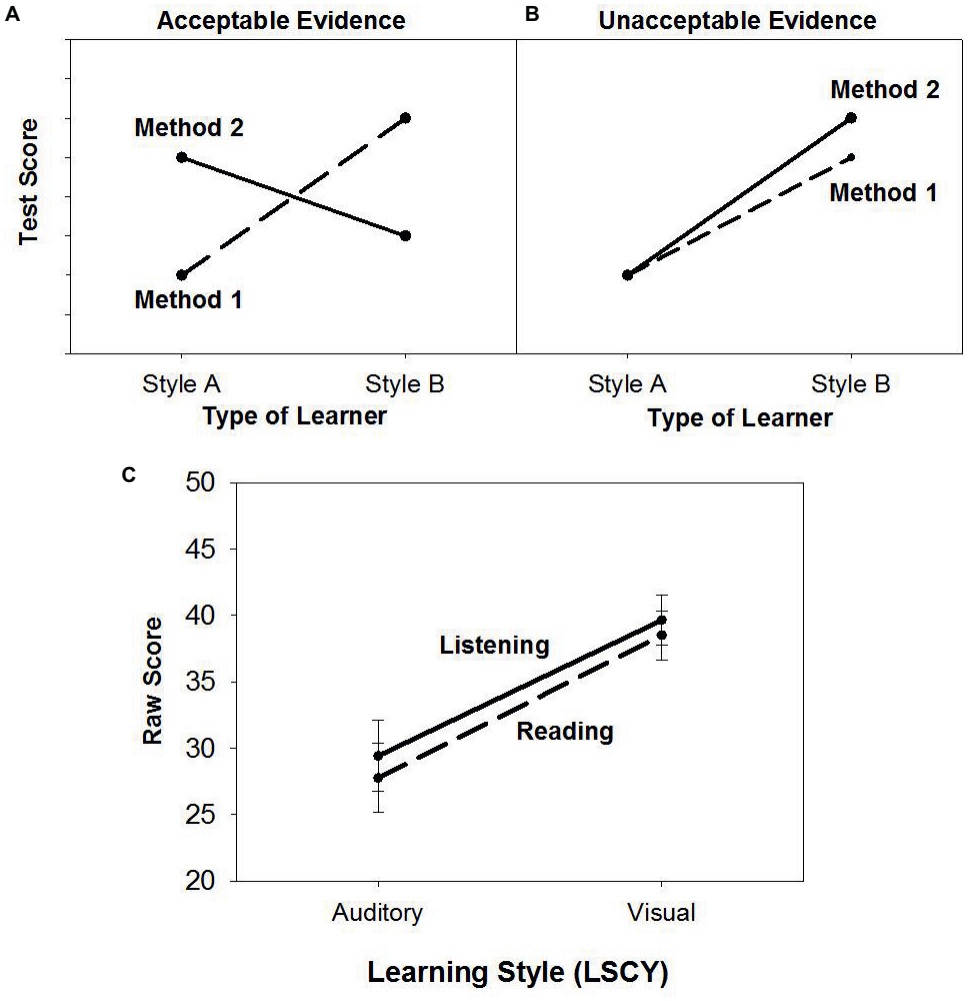
Graph **(A)** displays the pattern of evidence required to support the meshing hypothesis while Graph **(B)** displays one of several patterns of evidence that would constitute unacceptable evidence, as adapted from Pashler et al. (2009). Graph **(C)** displays the results from the current study, which show that there is no crossover effect. Bars represent standard errors. The 95% Confidence Interval for the L-AT ranged from 24.1 to 34.8 for students with an auditory learning style to 35.7–43.6 for students with a visual learning style. The 95% Confidence Interval for the R-AT ranged from 22.6 to 32.9 for students with an auditory learning style to 34.7–42.3 for students with a visual learning style.

According to Pashler et al. (2009), acceptable evidence supporting the meshing hypothesis would show a crossover between two learning style groups (auditory, visual) and listening and reading comprehension (L-AT, R-AT), as shown in Figure 2A. Figure 2B shows an example taken from Pashler et al. (2009) of one form of unacceptable evidence for the meshing hypothesis, where both auditory and visual learning style groups score higher on the same method, resulting in no crossover. Figure 2C shows the data from the current study. As shown in Figure 2C, contrary to the crossover pattern that would be expected to support the meshing hypothesis, the auditory and visual learning style groups *both* scored higher on listening comprehension than on reading comprehension making it unacceptable evidence.

### Limitations

This study was conducted using the entire population of 5th grade students present in one public middle school in rural Pennsylvania. Because all of the students attended the same school, they shared the same educational environment. Although the statewide-standardized reading assessment of the students in the study closely mirrors the results of the state (61.5% are proficient or above statewide and 60.3% of the students in the current study scored proficient or above), caution should be used when applying the findings to other populations.

Regardless of the mode of instruction, only text-based questions were used to test comprehension. By holding the format of the assessment constant, only one variable (instruction) was varied within the study. Text was chosen over a listening format because this is consistent with most assessments in the K-12 environment. However, it could be argued that using the same text-based format for the assessment favored those students who had a stronger visual learning style and, thus, masked evidence supporting the learning styles hypothesis. Indeed, visual learners performed significantly better than auditory learners on both the listening and reading comprehension tests. However, it should also be recalled that both learning style groups performed better on the listening comprehension test than the reading comprehension test, even though both were assessed with text-based questions. Irrespective of this potential limitation, it should be kept in mind that the critical test of the learning styles hypothesis rests in finding a significant *correlation* between learning style and learning achievement based on instruction. This was not found. Future studies may want to investigate the effect of the assessment format.

Additionally, the extent to which the results of this study can be generalized to other learning styles, forms of instruction, durations of instruction, and other types of material cannot be established.

## Discussion

It makes sense that visual learners would perform better when instruction is presented visually rather than auditorily. Likewise, it makes sense that auditory learners would perform better when instruction is presented in an auditory format rather than visually. This hypothesis had never been tested with K-12 students, making this study with 5th graders the first of its kind.

The results of this study add to the mounting evidence that does not support the widespread use of learning styles in the classroom. Most students, 68%, do not even have a clear learning style preference. For the ones who do, receiving instruction in their preferred style did *not* equate with better learning. Contrary to the expectations predicted by the learning styles hypothesis, we found (1) no significant positive relationship between auditory learning style and listening comprehension, (2) no significant positive relationship between visual learning style and reading comprehension, and (3) no differential effect of learning style on performance on a listening as compared to a reading comprehension test. Overall, matching instruction to meet a student’s auditory or visual learning style had no effect on student achievement. Teachers and schools should not devote time and resources to learning styles-based instruction.

Not only were we unable to support the learning styles hypothesis, we replicated a result with important implications for education. A main effect found that 5th graders with a preferred visual learning style performed significantly better than those with an auditory learning style on both listening and reading comprehension measures. This is similar to the results reported in a previous study with adults (Rogowsky et al. 2015). That is, both 5th graders and adults with a visual learning style had superior comprehension, regardless of instruction, while those with an auditory learning style scored significantly below their peers on *both* comprehension measures, regardless of instruction.

By 5th grade and beyond, an individual’s preference for auditory learning may reflect difficulty in learning to read or failure to become proficient. This would suggest that to achieve superior comprehension, which is vital for classroom learning, all students need as much opportunity as possible to build strong reading skills. Thus, contrary to the learning style hypothesis, it may be particularly important to focus on strengthening reading skills in all students, especially for auditory learners. That is, auditory learners may actually benefit more from additional instruction in their non-preferred modality. This is opposite to the specific recommendation prescribed by Rundle and Dunn to auditory learners: “Reading manuals, textbooks, articles, or documents is not your strength. You are strongly encouraged to utilize your strengths when learning new and difficult information” (Learning Styles, 2014).

Learning styles-based instruction was based on a theory that gained acceptance despite evidence. With the growing focus on the science of learning, current educational psychology journals and textbooks are making strides in drawing attention to the lack of evidence supporting learning styles-based instruction (Woolfolk, 2014). Unfortunately, many education textbooks continue to advocate for learning styles-based instruction and for teachers to use learning styles inventories and tests before planning instruction (Lynch, 2015). The results of this study add to a growing body of research that refutes the educational value of assessing and accommodating children’s learning style preferences with the goal of improving learning outcomes.

## Data Availability Statement

The datasets used in this study are available upon request to the corresponding author.

## Ethics Statement

The studies involving human participants were reviewed and approved by Bloomsburg University IRB Committee. Written informed consent to participate in this study was provided by the participants’ legal guardian/next of kin.

## Author Contributions

BR, the Principal Investigator, conducted literature review, wrote IRB, led meetings with school district administration and teachers where the research was conducted, oversaw onsite data collection over multiple visits, and worked closely with BC to analyze data, interpret and write-up the results for publication. BC led analysis of the data and writing up results for publication. PT provided expertise and ongoing consultation of study design, methodology, and analysis and reviewed and edited the final paper.

### Conflict of Interest

The authors declare that the research was conducted in the absence of any commercial or financial relationships that could be construed as a potential conflict of interest.
